# Visual field defect as the first manifestation of Alzheimer's disease

**DOI:** 10.1590/1980-5764-DN-2025-0292

**Published:** 2025-06-02

**Authors:** Marcelo Houat de Brito, Artur Martins Coutinho, Carla Rachel Ono, Eduardo Sturzeneker Trés, Sonia Maria Dozzi Brucki

**Affiliations:** 1 Universidade de São Paulo, Faculdade de Medicina, Hospital das Clínicas, Divisão de Neurologia, São Paulo SP, Brazil.; 2 Universidade de São Paulo, Faculdade de Medicina, Hospital das Clínicas, Divisão de Medicina Nuclear, São Paulo SP, Brazil.

**Figure 1:**
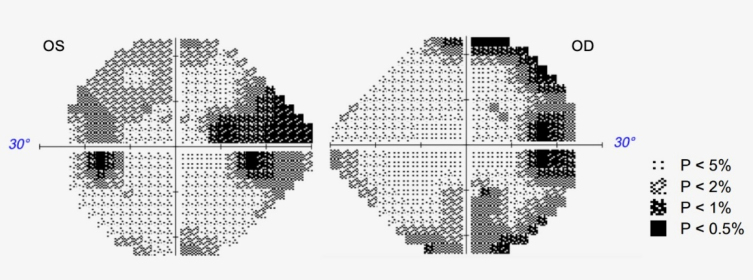
Computerized Visual Field Grayscale Map. Humphrey Visual Field Analyzer SITA-based central 24-2 threshold test demonstrating a bilateral peripheral visual field defect (macular sparing pattern), predominantly affecting the right hemifields. Fixation loss, false-positive, and false-negative response rates were within reliable limits.

**Figure 2:**
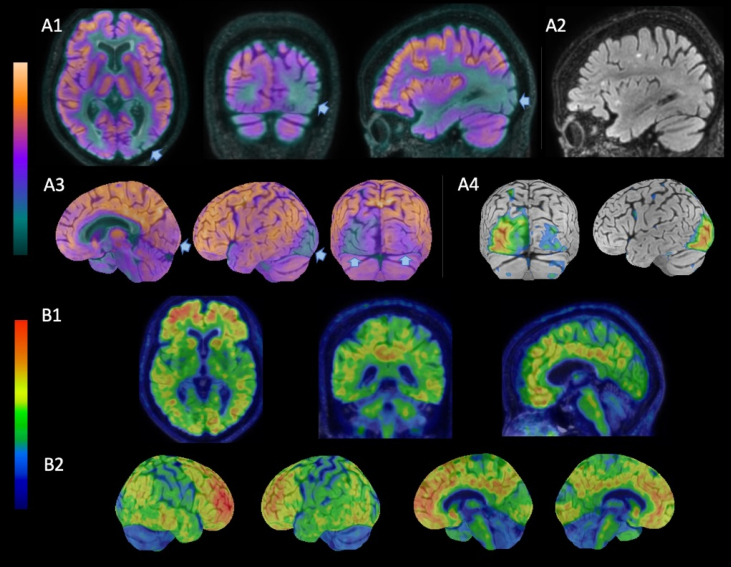
[18F]FDG and [11C] PiB–PET/MRI. (A1) [18F]FDG-PET/MRI: left-predominant bilateral hypometabolism in the occipital and temporo-occipital areas (arrows). (A2) Left sagittal MRI (FLAIR): no structural abnormalities detected. (A3) [18F]FDG-PET 3D-SSP images (Cortex ID suite, GE Healthcare). (A4) Z-score projection of hypometabolism. (B1) [11C]PiB-PET/MRI: diffuse cortical amyloid deposition – transaxial, coronal and sagittal views (B1) and 3D-SSP (B2).

A 73-year-old woman presented to the neurology clinic with memory issues persisting for the past two years. Cognitive and functional evaluation revealed amnestic mild cognitive impairment. Her medical history was notable for a slowly progressive visual field alteration over five years, which was previously investigated by ophthalmology through serial assessments of ocular pressure, optical coherence tomography, and brain and orbit magnetic resonance imaging (MRI), none of which identified any abnormalities. A new comprehensive investigation was performed, including a computerized visual field (Figure 1), which demonstrated a right-predominant bilateral peripheral visual field defect. Additionally, [18F] Fluordeoxyglucose (FDG) and [11C] Pittsburgh Compound B Positron Emission Tomography/Magnetic Resonance Imaging (PiB–PET/MRI) scans (Figure 2) revealed hypometabolism in the occipital lobes and cortical amyloid deposition, respectively.

These findings corroborate the diagnosis of a primary occipital (caudal) variant of posterior cortical atrophy (PCA), a rare atypical presentation of Alzheimer's disease. This variant is characterized by preferential involvement of the occipital lobes at onset, leading to greater impairment of the primary visual area and fewer visuospatial and perceptual deficits compared to the more common dorsal and ventral PCA^1,2^.
